# Reiter syndrome following protracted symptoms of Cyclospora infection.

**DOI:** 10.3201/eid0703.010317

**Published:** 2001

**Authors:** B A Connor, E J Johnson, R Soave

**Affiliations:** New York-Presbyterian Hospital and Weill Medical College of Cornell University, New York, New York, USA. bconnor@pol.net

## Abstract

Two large outbreaks of diarrheal illness associated with Cyclospora cayetanensis, a coccidian parasite, provided an opportunity to evaluate clinical syndromes associated with this enteric pathogen. Reiter syndrome, a triad of ocular inflammation, inflammatory oligoarthritis, and sterile urethritis, has been associated with enteric infections. We describe the first case of Reiter syndrome following protracted symptoms of Cyclospora infection.


### Reiter Syndrome Following Protracted Symptoms of *Cyclospora* Infection

**Bradley A. Connor, Erik Johnson, and Rosemary Soave**
New York-Presbyterian Hospital and Weill Medical College of Cornell University, New York, New York, USA

Two large outbreaks of diarrheal illness associated with *Cyclospora cayetanensis*, a coccidian parasite, provided an opportunity to evaluate clinical syndromes associated with this enteric pathogen. Reiter syndrome, a triad of ocular inflammation, inflammatory oligoarthritis, and sterile urethritis, has been associated with enteric infections. We describe the first case of Reiter syndrome following protracted symptoms of *Cyclospora* infection.

*Cyclospora*, a protozoan pathogen that causes a syndrome of diarrhea and fatigue, was responsible for two large-scale outbreaks in North America in 1996 and 1997 (1,2). These outbreaks, along with studies in *Cyclospora*-endemic areas (Nepal and Peru) and treatment of travelers returning from these areas, have increased our understanding of the clinical illness associated with this pathogen. Chronic diarrhea is not uncommon, especially in patients whose condition is untreated or partially treated. Recently, Guillain-Barré syndrome has been reported as an extraintestinal complication of *Cyclospora* infection (3). We describe Reiter syndrome diagnosed in a patient after prolonged gastrointestinal illness associated with *Cyclospora* infection.

### The Study

A 31-year-old man had onset of gastrointestinal illness on May 11, 1997, 7 days after attending a dinner at a country club. His symptoms (extreme fatigue, dizziness, fever of 101°F, intermittent vomiting and diarrhea) were similar to those of the 10 other guests at this party and caused him to miss work for 2 weeks. He visited his internist on May 22, 1997, and was hospitalized with predominant symptoms of fatigue and dehydration; he also reported constipation. Fluids and gentamicin were administered intravenously. Stool cultures and microscopy were negative; however, *Cyclospora* was not searched for specifically. After the patient was discharged from the hospital, diarrhea resumed, and a stool specimen was examined by our laboratory by a concentration technique and modified acid-fast staining. This examination identified *Cyclospora* oocysts. Standard therapy, trimethoprim/sulfamethoxazole, could not be administered because of the patient's allergy to sulfa, and as no effective alternative antibiotic therapy for *Cyclospora* infections exists, antibiotic treatment was not offered. Symptoms of epigastric pain and discomfort, bloating, and alternating diarrhea and constipation predominated, along with intermittent anorexia. An 8-pound weight loss (5% of body weight) was noted. By mid-June 1997, the patient's symptoms had abated slightly but were not completely resolved. He was enrolled at our institution in an open-label trial of albendazole, 400 mg 2 times a day for 14 days, and reported no change in his symptoms during this time. *Cyclospora* was present in stool after treatment with albendazole. Soft stool, intermittent diarrhea, and abdominal cramping persisted, along with proctalgia. Results of blood tests, including a complete blood count, biochemical profile, and liver function tests, were normal.

The patient's medical history included mild asthma with flare-ups approximately monthly, for which he had used theophylline. A diagnosis of prostatitis was made on clinical grounds in December 1996, 6 months before the onset of diarrheal illness and 10 months before the diagnosis of Reiter syndrome. These symptoms resolved with antibiotic therapy and did not recur.

When the patient first came to our institution on July 29, 1997, he was not in acute distress. His abdomen was soft and tender only to deep palpation in the left and right lower quadrants. There were no masses or enlarged organs. Stool specimens were negative for occult blood. As part of an ongoing study of small-bowel histopathologic changes associated with *Cyclospora* infections, the patient agreed to endoscopic evaluation. Upper gastrointestinal endoscopy was performed on August 15, 1997, with examination to the descending duodenum. This examination revealed inflammation of the distal esophagus and mild erythema of the gastric cardia and antrum, as well as erythema of the duodenal bulb and descending duodenum. Flexible fiber-optic sigmoidoscopy to the mid-descending colon was unremarkable. No *Cyclospora* oocysts were identified on either duodenal or stool aspirates. Biopsies of the duodenum revealed partial villous atrophy and moderate crypt hyperplasia with increased intraepithelial lymphocytes and rare intraepithelial neutrophils. Biopsies of the gastric antrum and cardia were normal. A rapid urease test performed on the gastric biopsy was negative for *Helicobacter pylori*. Electron microscopy evaluation of the small bowel biopsies revealed acute and chronic inflammation evidenced by focally intense epithelial injury, including vacuolization, lipid accumulation, and abundant interstitial and epithelial reactive elements. Sigmoid and rectal biopsies revealed no histopathologic changes.

Gastrointestinal symptoms persisted, including abdominal bloating and cramping and intermittent soft stool, along with fatigue. In September 1997, the patient noted pain and

Address for correspondence: Bradley A. Connor, 50 East 69th Street, New York, NY 10021; fax: 212-734-4200; e-mail: bconnor@pol.net soreness in knees, ankles, right first toe, and plantar heels. During early October 1997, he noted a constellation of symptoms, including left eye pain, dysuria, arthralgias, and a painful ulcer of the right buccal mucosa. An ophthalmologist confirmed that the left eye pain resulted from iritis and episcleritis. A rheumatologist suspected the diagnosis of Reiter syndrome on the basis of the constellation of findings and negative serologic tests for Lyme disease and *Chlamydia*. A urinalysis was negative for leukocytes and bacteria, although no attempts were made to recover *Chlamydia* from the urine. Stool microscopy was negative for parasites, and a stool culture was negative for bacterial pathogens. The patient was noted to be negative for HLA-B27. Oral corticosteroids were begun for the iritis symptoms, and he was treated with doxycycline (100 mg twice a day) for its antiinflammatory effects. The arthritis symptoms improved promptly and dramatically. Four days after beginning doxycycline therapy, the patient complained of progressively severe odynophagia, and a clinical diagnosis of pill-induced esophagitis or ulcer was made. The symptoms resolved with a combination of sucralfate suspension and omeprazole, and the patient has remained clinically well.

### Conclusions

Reiter syndrome, characterized by the triad of ocular inflammation (conjunctivitis, iritis, episcleritis), inflammatory oligoarthritis, and sterile urethritis, usually occurs several weeks after a triggering infection (4). Infections associated with Reiter syndrome include genitourinary *Chlamydia* or enteric infections with *Salmonella*, *Shigella*, *Yersinia*, *Campylobacter*, *Clostridium difficile*, and *Cryptosporidium*, although the incidence of Reiter syndrome with each of these infections is suspected to be low. This is the first reported case of Reiter syndrome following *Cyclospora* infection. Although Reiter syndrome in this patient could have been coincidental, we propose *Cyclospora* as another infectious trigger for Reiter syndrome.

This patient's history is unique in several respects. Because of sulfa allergy, he was inadequately treated for the *Cyclospora* infection. An experimental trial of albendazole, which is used to treat other parasitic infections, did not shorten his illness. Chronic gastrointestinal symptoms persisted for 12 weeks before endoscopy, when variable crypt hyperplasia and villous atrophy were noted—characteristic findings in small bowel biopsies of patients with *Cyclospora* infection (5). No *Cyclospora* oocysts were noted on stool examination at the time of endoscopy. The pathogenesis of Reiter syndrome may involve molecular mimicry between microbial fragments in synovial fluid and the HLA-B27 molecule; however, it is unknown whether microbial fragments of *Cyclospora* could be found in synovial fluid, and in this case the patient was HLA-B27 negative (6). Inflammatory lesions in the small intestine may allow antigen priming of receptive T lymphocytes and trigger an inflammatory response in affected organs. Depending on the length of this process, it can be hypothesized that prompt, effective treatment of *Cyclospora* infections may minimize the risk for postinfectious complications such as Reiter syndrome.

Dr. Connor is clinical associate professor of medicine at Weill Medical College of Cornell University, associate attending physician at New York-Presbyterian Hospital, and adjunct faculty member at Rockefeller University. He was part of the Kathmandu, Nepal, team that first described the illness associated with *Cyclospora* and has done research on the pathogenesis, clinical illness, epidemiology, and treatment of *Cyclospora* infections.

### References

1. Herwaldt BL, Ackers ML, and the Cyclospora Working Group. An outbreak in 1996 of Cyclosporiasis associated with imported raspberries. N Engl J Med 1997;336:1548-56.
2. Herwaldt BL, Beach MJ, and the Cyclospora Working Group. The return of *Cyclospora* in 1997: Another outbreak of cyclosporiasis in North America associated with imported raspberries. Ann Intern Med 1999;130:210-20.
3. Richardson RF, Remler BF, Katirji B, Murad MH. Guillain-Barré syndrome after *Cyclospora* infection. Muscle Nerve 1998;21:669-71.
4. Konttinen YT, Nordstrom DC, Bergroth V, Leirisalo-Repo M, Santavirta S. Occurrence of different ensuing triggering infections preceding reactive arthritis: a follow-up study. BMJ 1988;296:1644-5.
5. Connor BA, Shlim DR, Scholes JB, Rayburn JL, Reidy J, Rajah R. Pathologic changes in the small bowel in nine patients with diarrhea associated with a coccidia-like body. Ann Intern Med 1993;119:377-82.
6. Hermann E, Yu DT, Meyer zum Buschenfelde KH, Fleischer B. HLA-B27 restricted CD8 T-cells derived from synovial fluids of patients with reactive arthritis and ankylosing spondylitis. Lancet 1993;342:646-50.
